# Chemical Analysis of Suspected Unrecorded Alcoholic Beverages from the States of São Paulo and Minas Gerais, Brazil

**DOI:** 10.1155/2015/230170

**Published:** 2015-10-01

**Authors:** Giuseppina Negri, Julino Assunção Rodrigues Soares Neto, Elisaldo Luiz de Araujo Carlini

**Affiliations:** ^1^Brazilian Information Center on Psychotropic Drugs (CEBRID), Department of Preventive Medicine, Federal University of São Paulo (UNIFESP), Botucatu Street 740, 04023-900 São Paulo, SP, Brazil; ^2^Brazilian Information Center on Psychotropic Drugs (CEBRID), Department of Psychobiology, Federal University of São Paulo (UNIFESP), Botucatu Street 586, 04023-900 São Paulo, SP, Brazil

## Abstract

Our study analyzed 152 samples of alcoholic beverages collected from the states of São Paulo and Minas Gerais, Brazil, using gas chromatography with flame ionization detection (GC-FID) and mass spectrometry (GC-MS), Fourier transform infrared spectroscopy (FT-IR), and inductively coupled plasma atomic emission spectrometry (ICP-AES). The methanol content varied from 20 to 180 ppm in 28 samples, and the limit of the accepted level of 200 ppm was exceeded in only one sample. High content of cyanide derivatives and ethyl carbamate, above the accepted level of 150 ppb, was observed in 109 samples. Carbonyl compounds were also observed in 111 samples, showing hydroxy 2-propanone, 4-methyl-4-hepten-3-one, furfural, and 2-hydroxyethylcarbamate as main constituents. Copper was found at concentrations above 5 ppm in 26 samples; the maximum value observed was 28 ppm. This work evaluated the human health risk associated with the poor quality of suspected unrecorded alcohols beverages.

## 1. Introduction

Public health problems caused by excessive consumption of alcoholic beverages are a serious concern in many countries [1], and Brazil is no exception [2–5]. This situation may worsen, due to the sale of unrecorded alcoholic beverages. According to World Health Organization [1] “unrecorded” is an overview category that includes any alcohol not taxed as alcoholic beverage or registered in the jurisdiction where it is consumed.

Cachaça is a beverage exclusive to Brazil, having an alcohol content of 38–48% v/v at 20°C; which is obtained by distilling fermented sugarcane juice and has particular sensory characteristics [6]. During the alcoholic fermentation of sugarcane with wild, unselected yeasts, ethanol and carbon dioxide are formed as major products [7, 8].

However, an excessive amount of by-products considered as contaminants may be present in alcoholic beverages from several countries, when the production process, such as distillation is not carried out in accordance with quality control [7, 9]. For example, high concentrations of ethyl carbamate (EC) [9–11] have been found in alcoholic beverages, especially in spirits derived from cyanogenic plants, such as cachaça derived from sugarcane [11] and tiquira from Cassava (*Manihot esculenta*) [4]. Cyanide derivatives can be formed through the thermal cleavage and enzymatic reaction of cyanogenic glycosides found in these plants [4, 9–11]. Other by-products found in cachaça are higher alcohols with 3–5 carbon atoms [12–14], ethyl esters [15], acetates [16, 17], organic acids [8], and carbonyl compounds, such as 5-hydroxymethylfurfural and furfural [6].

In Brazil, good manufacturing practices and quality control for alcoholic beverages are regulated by the Ministry of Agriculture, Livestock and Supply (MAPA). According to research carried out by Minas Gerais government, about 95% of alembics are informal; that is, 100 million among the 200 million liters of beverages produced in Minas Gerais are unrecorded [18]. According to a study carried out by Getúlio Vargas Foundation (FGV), it is estimated that the beverage consumed in Brazil was 13,6 billions of liters and the unrecorded beverages correspond to 20.3% [19].

Brazil ranks 84th in the Human Development Index (HDI: 0.718) [20]. Its total population in 2010 was 190,755.799 inhabitants, with a population density (PD) of 22.4 inhabitants/km^2^ and a life expectancy of 73.48 years [20]. The city of São Paulo has a PD of 7,387.69 inhabitants/km^2^ and a municipal human development index (HDI) of 0.841 [21], and Diadema has a PD of 12,519.10 inhabitants/km^2^ and an HDI of 0.79 [21]. In Minas Gerais State, the selected cities have the following. Salinas has a PD/HDI: 20.75/0.699, Belo Horizonte has a PD/HDI: 7,167.02/0.839, Patrocínio has a PD/HDI: 28.69/0.799, Passa Quatro has PD/HDI: 56.21/0.832, and Itanhandu has PD/HDI: 98.87/0.795 [21].

The downside of the excessive alcohol consumption by Brazilians was observed in different individual and social aspects of the society, such as unemployment, increased violence, increase in venereal diseases, and AIDS. According to Nappo and Galduróz [22] the presence of alcohol was detected in the blood of 15.2% of the deceased persons examined at the Institute of Forensic Medicine from São Paulo city between 1987 and 1992. Unrecorded alcoholic beverages can be considered an aggravating factor to this problem. Thus, as a measure to reduce the harmful use of alcohol, one of the goals emphasized by the World Health Organization [1] is the need to reduce the impact of alcohol from informal origin on public health through the adoption of appropriate measures. Therefore, it is pertinent to carry out the chemical analyses of unrecorded beverages to protect the health of Brazilian people. This paper describes the results of chemical analyses of alcoholic beverages deemed unrecorded from the states of São Paulo (SP) and Minas Gerais (MG).

## 2. Experimental Procedures

### 2.1. Collection

Sixty-five samples of alcoholic beverages were collected from two cities in the state of São Paulo (SP), and 87 from five cities in Minas Gerais, from 2010 to 2012, for a total of 152 samples. In São Paulo state 25 samples were collected in São Paulo city and 40 in Diadema city. In Minas Gerais State 9 samples were collected in Salinas, 18 in Belo Horizonte, 22 in Patrocínio, 19 in Passa Quatro, and 19 samples in Itanhandu. They were collected from homes, from bars, and at parties. In Brazil there are many popular parties, such as “rodeio,” “baladas,” and “carnaval,” where the unrecorded beverages have very high consumption. The criteria that drove the collection of the samples were the absence of labels or the absence of MAPA registration on the label, no tax seal, low price, or inadequate packaging.

This research was approved by Committee of Ethics in Research of UNIFESP (CEP N° 0195/12).

### 2.2. Chemical Analyses

The chemical analyses were carried out on 114 samples of cachaça, 18 of whiskey, 9 of liqueur, 7 of vodka, 2 of tequilas, 1 of wine, and 1 of beer. The methodologies used in these analyses were based on the AMPHORA (Alcohol Measures for Public Health Research Alliance) methodologies, which were developed for analyses of unrecorded alcohols [23–26].

#### 2.2.1. Analysis of Methanol and Higher Alcohols Using Gas Chromatography with a Flame-Ionization Detector (GC/FID)

The quantification of methanol and higher alcohols was performed by GC/FID [27] using a Shimadzu gas chromatography, GC-17A model, equipped with a DB 624 capillary column (30 m × 0.55 mm × 0.25 *μ*m). The temperatures of the detector and injector were set at 250°C, the injection mode was set to a flow split of 1 : 25, and the injection volume was set to 1.00 *μ*L of sample. The column oven temperature was programmed with an initial temperature of 40°C, which was maintained during 5 min. The temperature was increased to 250°C at a rate of 10°C/min and was kept for 10 min. Compounds were identified by comparing the retention times to those of analytical standards. The standard curves for methanol and higher alcohols were plotted using 10, 20, 40, 80, 100, 150, 200, and 250 *μ*g of standard alcohols dissolved in 100 mL anhydrous alcohols as external standards. Chromatographic analysis of each standard solution was performed three times, and the calibration curve was plotted as the peak area of the chromatogram on the *y*-axis against that of the standard compound (*μ*g) on the *x*-axis [26, 27]. The data points were fitted to a best-fit line using the linear regression method.

#### 2.2.2. Analysis of Ethanol, Cyanide Derivatives, and Carbonyl Compounds Using Fourier Transform Infrared Spectroscopy (FT-IR)

**Figure 1:**
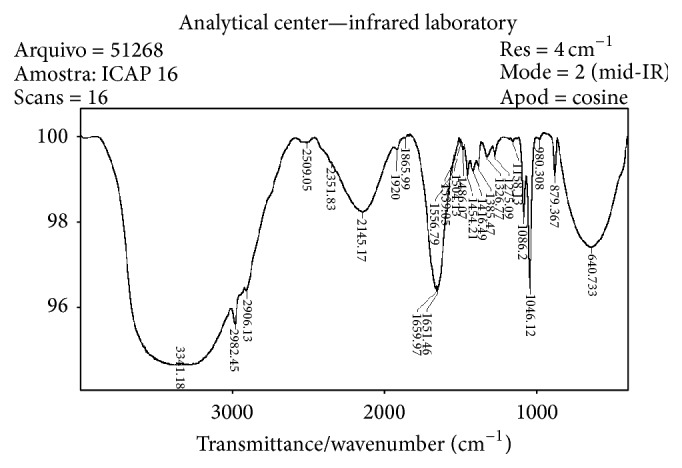
IR spectrum of a sample of Brazilian cachaça showing the main bands: at 3341–2906 cm^−1^ attributed to O–H stretching of alcohols, water, carboxylic acids, and amides; at 2145 cm^−1^ attributed to the asymmetric vibration of NCO^−^ in cyanate-copper complexes; at 1659–1651 cm^−1^ attributed to CO stretching vibrations conjugated with double bonds (C=C) adjacent to a carbonyl group; at 1086 and 1046 cm^−1^ attributed to stretching vibrations of hydrogen-bonded C–OH alcoholic groups.

FT-IR spectra were recorded at room temperature (ca. 25°C) using a Bomem spectrometer by scanning over the frequency range 4000–400 cm^−1^ at a resolution of 5 cm^−1^. Ethanol shows three bands: an intense band at 1046 cm^−1^ and two other bands of medium intensity, centered at 1086 and 879 cm^−1^, respectively [23, 28]. The ethanol quantification was carried out using the analytical band at 1046 cm^−1^ (Figure 1) in the FT-IR. For the determination of ethanol content (percent by volume—%vol.), linear regression analysis of the relative peak absorption versus concentration for standard ethanol/water solutions with ethanol contents of 10%, 20%, 30%, 40%, 50%, and 60% was used in order to obtain calibration curves [23, 28]. Beverage alcoholic samples that showed turbid were filtered to prevent disturbances in the optical path length of the cuvette. Under the experimental conditions, the analytical signal increased linearly with the ethanol concentrations. The precision of the procedure was estimated by average concentration of three FT-IR determinations of each sample. The results among the three determinations for each sample did not exceed 1.1%. The results obtained using FT-IR were correlated with those obtained using GC-FID and showed similar values.

Cyanide derivatives and carbonyl compounds in alcoholic beverages show absorptions in the FT-IR spectra due to various functional groups [23, 28]. Thus, this analysis was also important for assessing the presence of carbonyl compounds and cyanide derivatives, which are precursors of EC.

#### 2.2.3. Copper Analysis Using Inductively Coupled Plasma Atomic Emission Spectrometry (ICP-AES)

The copper content was analyzed using Spectro Arcos SOP-FHS12 equipment. The samples were diluted with a 1% solution of nitric acid and ethanol (4%, v/v), which were added to the calibration solution [25–27]. The operating conditions were frequency 38 MHz; double diffraction net 352 line mm^−1^; generator 1280 W; plasma was formed in a stream of argon gas of 13 L min^−1^; and cone spray nebulizer pressure 58 psi.

#### 2.2.4. Analysis of Carbonyl Compounds in Two Samples of Cachaça Using Gas Chromatography Mass Spectrometry (GC/MS)

Two unrecorded beverage samples that showed high concentrations of carbonyl compounds were analyzed by GC-MS with the aim of identifying the carbonyl compounds that produced the band at 1651–1659 cm^−1^ (attributed to CO stretching vibrations conjugated with double bonds adjacent to a carbonyl group) in the FT-IR spectra (Figure 1). This analysis was carried out using a Shimadzu GCMS-QP505A gas chromatography coupled to a quadrupole mass selective spectrometer. The chromatographic conditions were an injection mode with a flow split of 1 : 25 with an injection volume of 1.00 *μ*L of sample. The separation was performed using a DB 624 capillary column (30 m × 0.55 mm × 0.25 *μ*m). The column oven temperature was programmed with an initial temperature of 40°C, which was maintained during 5 min. The temperature increased to 300°C at a rate of 10°C/min and was kept for 10 min. MS analyses were made in the electron impact (EI) mode (70 eV). Helium was used as the carrier gas with a flux of 1.5 mL min^−1^ and a split ratio of 100 : 1; linear velocity of 63 cm/sec, total flow 77.3 mL/min., and solvent cut time of 3.0 min. The MS conditions were filament current, 0.3 mA; detector voltage, −0.7 kV, ion source temperature, 300°C; interface temperature, 300°C; scan speed 2 scans s^−1^. The mass range was from* m*/*z* 29–300 *μ* and the chromatogram was acquired in total ion current (TIC). The carbonyl compounds were identified through comparison of their mass spectra with those reported in the GC-MS computer database (Wiley 275, Wiley 229, and NIST 21) and literature data. Beside this, standard compounds, such as formic acid, acetic acid, and furfural were coinjected in order to confirm the identification of compounds. The determination of its relative amounts was based on the regions under the corresponding chromatogram peak.

## 3. Results

Among the 65 samples collected from SP (Table 1), only 21 had labels. Furthermore, only 12 displayed a MAPA registration number. Similar results were obtained for the 87 samples collected from MG, all of which showed signs of being unrecorded, with 49 unlabeled. Only one sample displayed a MAPA registration number. There were no significant differences between the results obtained from the two states. Therefore, all data were pooled, and only those for which concentrations exceeded the legally admissible limits were emphasized.

### 3.1. Methanol and Higher Alcohols Analyzed by GC/FID

Methanol was quantified in only 54 of the 65 samples collected in SP, due to impurities present in the remaining samples, methanol, around 80 ppm, was detected in three cachaça and one liqueur samples. The wine sample showed a methanol content of 240 ppm, which is above the legal limit of 200 ppm (Table 2). In MG, methanol was detected in 24 samples, of which 19 were cachaça with levels ranging from 42 to 169 ppm, one vodka (180 ppm), one tequila (120 ppm), and three whiskey samples, one with 26 ppm and two with 79 ppm (Table 2), all below the legal limit of 200 ppm.

In the samples collected from SP, the higher alcohols found are 2-butanol,* n*-butanol,* n*-propanol, and isoamyl alcohol, with levels ranging from 2 to 40 ppm, in 47 of the 54 samples analyzed. In MG, 2-butanol,* n*-butanol,* n*-propanol, and isoamyl alcohol were found in 84 of the 87 samples with levels also ranging from 2 to 40 ppm, which is below the legal limit of 360 ppm.

### 3.2. Ethanol, Cyanide Derivatives, and Carbonyl Compounds Analyzed by FT-IR

Ethanol content reported as the “percent by volume” (% vol) was determined using two different methods GC-FID and FT-IR [23–28]. However, as GC/FID and FT-IR showed similar yields, the results of ethanol content were based on the FT-IR spectroscopy data. Ethanol shows three bands: an intense band at 1046 cm^−1^ and two other bands of medium intensity, centered at 1086 and 879 cm^−1^, respectively. These bands are due to vibrational transitions of the C–O–H system: C–O stretching vibration (*p*-OH) and O–H bending vibration out of plane [25, 29]. The ethanol quantification was carried out using the analytical band at 1046 cm^−1^ (Figure 1) in the FT-IR. Only 32 out of 65 samples collected from São Paulo had ethanol contents above 38% (Table 2), which are in accordance with the legal limit. In 11 samples, the ethanol content was lower than 38% (20% to 38%), while in the other 22 the ethanol content varied from 5% to 20% (Table 2). In MG, 36 of the 87 samples analyzed contained an ethanol content above 38%, in 13 samples less than 38% (20% to 38%) was detected, while for the other 38 samples, the ethanol content varied from 5% to 20%.

The presence of ethyl carbamate (an ester of carbamic acid) was observed through its cyanide derivatives precursors, the hydrocyanic acid (HCN) and cyanic acid (HCNO and its tautomeric form HOCN). The FT-IR spectra of various samples of unrecorded beverages showed a large band in 2145 cm^−1^ that was attributed to the asymmetric vibration of the cyanate (NCO)^−^ in the cyanate-copper complexes [10, 11, 29, 30], as can be seen in Figure 1. According to Baffa Junior et al. [10], the formation of EC from HCN is based on the complexation of HCN to copper, followed by its oxidation to HCNO, which reacts with ethanol to form EC. Copper acts as an important catalyst in the conversion of HCN into EC in cachaça [29, 30].

In SP, 24 of the 65 tested samples showed the presence of cyanide derivatives, while in MG, cyanide derivatives were present in 85 out of 87 samples. It is interesting to mention that among the samples from SP, the presence of cyanide derivatives was observed only in cachaças mixed with plants used in folk medicine of Brazil, whereas in the samples from MG, this contaminant was found in almost all samples, including whiskey and vodka. In SP, 26 out of the 65 samples tested showed a high content of carbonyl compounds, while in MG, a high content of carbonyl compounds was present in 85 out of 87 samples.

### 3.3. Copper Content Using Inductively Coupled Plasma Atomic Emission Spectrometry (ICP-AES)

In the samples of cachaça from SP, the copper content ranged from 1.0 to 28.0 ppm, with 11 samples exhibiting copper contents ranging from 5.0 to 28.0 ppm, upper limit 5.0 ppm. Out of these, only one with a copper content of 7.72 ppm displayed the MAPA registration number. In samples from MG, the copper content ranged from 1.0 to 26.0 ppm, with 15 samples exhibiting copper contents ranging from 5.0 to 28.0 ppm (Table 2). This is different from the results for the whiskey, tequila, and vodka samples, with average within the normal level, lower than 5 ppm.

### 3.4. Analysis of Carbonyl Compounds in Two Samples of Cachaça Using GC-MS

**Figure 2:**
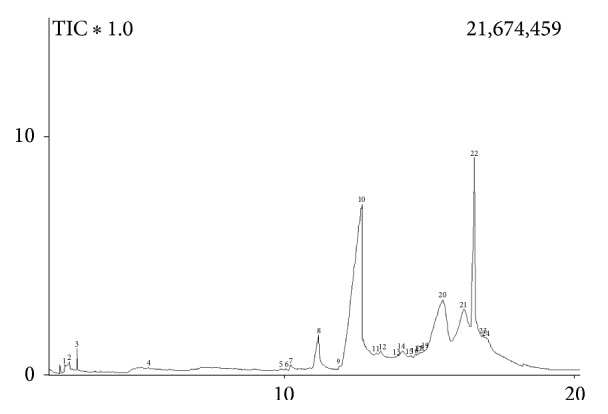
Total ion chromatogram obtained in the analyses of cachaça sample from Minas Gerais through GC-MS. The numbers corresponding to the following compounds. 1: formic acid. 2: acetic acid. 3: hydroxy 2-propanone. 8: furfural, 10: 4-methyl-4-hepten-3-one. 20: 2-hydroxyethylcarbamate, 22: 5-hydroxymethyl furfural (5-HMF).

Carbonyl compounds present in cachaça were identified in the analyses of two samples of cachaça, which showed a high content in FT-IR analyses, using GC-MS, and the results are shown in Table 3. The carbonyl compounds were identified through comparison of their mass spectra with those reported in the GC-MS computer database (Wiley 275, Wiley 229, and NIST 21), standard compounds, such as formic acid, acetic acid, and furfural and literature data [31]. The obtained chromatogram is shown in Figure 2. The Mass Spectrometry analyses were carried out in the electron impact (EI) mode (70 eV). The main constituent found in these two cachaças samples was proposed as being 4-methyl-4-hepten-3-one, which showed a molecular ion at* m/z *126 (C_8_H_14_O) (Table 3). The presence of this unsaturated ketone was corroborated by the band at 1656 cm^−1^ observed in FT-IR, as can be seen in Figure 1. The presence of the 5-hydroxymethylfurfural (5-HMF) was detected mainly through the base peak at* m*/*z *81, which was attributed to the ion (C_5_H_5_O)^+^ formed by furan ring with a methyl group, through comparison with literature data [32].

The presence of 2-hydroxyethylcarbamate [11, 33] was observed through the molecular ion at* m/z* 105 and base peak at* m/z* 60, which could be attributed to the ion (CH_2_NO_2_) indicating the presence of carbamate moiety. Carbamates derivatives had been studied through Tandem mass spectrometry measurement [33].

## 4. Discussion

The unrecorded beverages have attracted the attention of WHO, due to the presence of compounds such as methanol and ethyl carbamate and its precursors, furfural, 5-hydroxymethylfurfural, acrolein, and other toxic compounds. There have been discussions on whether or not these substances can produce serious illness effects in humans [24, 25, 34, 35]. Many studies were carried out to evaluate the chemical composition of unrecorded alcohol from Nigeria, Lithuania, Hungary, Poland, Guatemala, Vietnam, and Brazil, with the aim to investigate the possible health impact of unrecorded alcohol [24, 25, 34, 35]. In Brazil, Nagato et al. [36] carried out the analyses of methanol, ethanol, and higher alcohol content in 608 samples of alcoholic beverages confiscated by the police, from 1993 to 1999. Among them, 391 were counterfeit, being the ethanol content below the accepted levels in all of them. In two of the samples, high methanol content (14 g/100 mL and 10 g/100 mL) was detected, which is much above the accepted level of 0.02 g/100 mL.

In our study, the contamination with methanol, higher alcohols, and copper was analyzed in 152 samples of unrecorded alcoholic beverages from SP and MG. Beside this the contamination with cyanide derivatives and carbonyl compounds, as well as the content of ethanol, was also evaluated. A similar extensive study was carried out in Russia involving 81 samples of unrecorded alcohol [37].

As shown in Table 1, the missing labels, the absence of tax seal, MAPA registration number, inadequate packaging, and low price indicated that the samples collected show clear evidence of being unrecorded, which was corroborated by the results of chemical analysis (Tables 2 and 3). Many of the beverages exhibited ethanol contents lower than 38%, which was also observed by other authors [6–8, 38] in the analyses of Brazilian cachaça. Low alcohol levels could be related to the conditions during storage of the beverages, such as the temperature, humidity, and porosity of the barrel [6–8, 38].

Methanol is the most toxic alcohol, with documented cases of poisonings, including optic nerve damage and fatal intoxications [8, 38, 39]. In our sample, the methanol levels were below the 200-ppm limit for all unrecorded beverages, except one wine sample, which is consistent with several analyses of cachaça samples collected from southern Minas Gerais and other regions of Brazil [6–8, 38, 39]. The incidence of methanol poisoning was infrequent but had received high media exposure [24–27]. Higher alcohols have also been found in low levels as reported by other authors [12]. Higher concentrations of methanol, isobutanol, 1-propanol, and isoamyl alcohol were found in illicitly distilled spirits from Hungary [35]. High amounts of these alcohols could cause hepatic damage, contributing to the high level of alcohol-induced liver cirrhosis [40].

Ethyl carbamate (EC) is a carcinogen (group 2A), and its legal limit is 150 ppb [11]. EC is formed from the reaction of ethanol and compounds containing carbamoyl groups. The main EC precursors are commonly generated from arginine metabolism by* Saccharomyces cerevisiae* or lactic acid bacteria, which is accompanied by the fermentation process [10, 11, 41, 42]. High EC content, reaching levels as high as hundreds of micrograms per liter, was detected in Brazilian sugarcane spirits [42]. Such high concentrations were found in samples from the state of Minas Gerais and Pernambuco in many authors [10, 11, 29, 30, 41, 42].

In destillation process of cachaça, the head fraction comprises compounds such as methanol, acetaldehyde, and ethyl carbamate with more solubility in ethanol than in water. The heart fraction comprises mainly ethanol and higher alcohols, while the tail fraction comprises compounds such as acetic acid and 5-hydroxymethylfurfural (HMF), which are less volatile than ethanol [43]. In Brazil, most producers used direct-fire alembic, in which the heat produced by burning sugarcane bagasse reaches temperatures near 100°C. In this temperature, the fermented sugarcane formed an azeotropic mixture consisting of ethanol and water, whose boiling point is below 100°C [43]. Although this temperature is below the EC boiling point (186°C), it does not prevent that cyanide derivatives, hydroxymethylfurfural, furfural, and ethyl carbamate will be carried out to the distillate [11, 43]. In addition, this system used to heat the stills does not provide a constant rate of heat transfer [11].

The contamination with ethyl carbamate was also found in many spirits from Hungary, Poland, and other European countries [24–27, 34, 35]. This problem occurs when the homemade beverages are produced from fruit materials or medicinal plants that contained cyanogenic glycosides, without the application of measures to prevent this contamination [24–27]. Cachaça distilled in alembics contains a higher content of EC, due to the dissolution of the basic copper carbonate from the inner wall of the still by the acid vapors formed, during the distillation process, which cause the corrosion and release of copper into the beverages, favoring EC formation [6, 11, 42]. Generally, sugarcane spirits produced in pot still distillation had lower EC values than those from continuous distillation columns [11].

Cachaça may contain considerable concentrations of As, Pb, and Cu, which are harmful for human health if ingested in high quantities. Cachaça is distilled in copper stills and copper contamination can take place. High accumulation of copper in the organism could cause Wilson's disease [44] and consequently toxic effects, because copper, is not excreted by the liver. This disease, if untreated, can lead to brain and liver damage. Copper catalyzes the formation of cyanate, which react with ethanol to form ethyl carbamate [10, 11]. The Brazilian law establishes a limit for copper content of 5 ppm, prohibits the use of copper stills for the production of alcoholic beverages, and requires the use of stainless steel stills. Our study found 26 cachaças (11 from SP and 15 from MG) with high copper levels (Table 2), consistent with the results obtained by other authors [6, 11, 29, 30].

Various aldehydes, formaldehyde, acetaldehyde, propionaldehyde (acrolein), furfural, and 5-hydroxymethylfurfural (5-HMF), together with formic acid and acetic acid, are obtained as by-products during the production of cachaça [6, 24–27, 38]. 5-HMF is cytotoxic in high concentrations and irritating to the eyes, upper respiratory tract, skin, and mucous membranes [45]. As far as we know among the compounds listed in Table 3, hydroxy 2-propanone, 4-methyl-4-hepten-3-one, and 2-hydroxyethylcarbamate were not previously reported in cachaça.

## 5. Conclusion 

This study revealed the following facts. (1) In the Brazilian market, alcoholic beverages are present without MAPA registration number, unlabeled, and with suspiciously low price. These unrecorded alcoholic beverages are certainly acquired and consumed by the population. (2) Many of these unrecorded beverages have low alcohol content and consequently high water content. (3) Chemical assays showed the presence of various highly toxic contaminants, mainly cyanide derivatives. Therefore, the consumption of these unrecorded alcoholic beverages may be an aggravating factor that adversely affects the health of Brazilians people. (4) Adequate sanitary and legal measures should be taken to correct these problems. (5) More severe quality control of beverages should be carried out according to WHO. (6) Further, the population should be informed about the risk of consumption of unrecorded beverages.

## Figures and Tables

**Table 1:** Number of samples among the 152 samples of alcoholic beverages collected from the states of São Paulo (number of samples 65) and Minas Gerais (number of samples 87), Brazil, which are unlabeled and did not display a MAPA registration number or tax seal.

Items considered	Samples from São Paulo (*N* = 65)	Samples from Minas Gerais (*N* = 87)
Missing label	44	49
MAPA registration number	12	1
Tax seal	0	24
Inadequate packaging	2	1
Low price	7	12

**Table 2:** Results obtained in chemical analyses carried out in 65 samples of alcoholic beverages from state of São Paulo^*^ and 87 from state of Minas Gerais, Brazil. Methanol content (limit 200 ppm), ethanol content in percentage by volume (38% to 48%), and copper (limit 5 ppm).

States	Number of samples	Methanol (ppm)			Ethanol (%)			Copper (ppm)	
		<20	20 to 180	>240	>38	20% to 38%	5% to 20%	<5	5 to 28
São Paulo	65^*^	49	4	1	32	11	22	54	11
Minas Gerais	87	63	24	0	36	13	38	72	15

^*^For analyses of methanol content in samples from São Paulo State, among the 65 samples, 11 were not analyzed, due to impurities.

**Table 3:** Carbonyl compounds identified through GC-MS analysis in two Brazilian cachaça samples.

Retention time	EI-MS data (*m*/*z*)^*^ (percentage)	Proposed structure	Peak percentage^**^
2.2	46 (100), 45 (80)	Formic acid	2%/3%
2.6	60 (80), 45 (100), 43 (90)	Acetic acid	4%/2%
2.8	74 (1), 43 (100), 31 (40)	Hydroxy 2-propanone	4%/3%
11.2	96 (20), 95 (100), 39 (60)	furfural	4%/6%
12.6	126 (1), 97 (90), 69 (60), 41 (100), 39 (80)	4-Methyl-4-hepten-3-one	32%/29%
15.43	105 (1), 73 (60), 60 (100), 43 (70)	2-Hydroxyethylcarbamate	19%/40%
16.5	126 (1), 97 (5), 82 (50), 81 (100), 53 (50), 39 (40)	5-Hydroxymethyl furfural (5-HMF)	28%/6%

^*^Data obtained (molecular ions and fragments) by mass spectrum analysis.

^**^Percentages determined by peak areas in the chromatograms.

## References

[1] WHO (2004). *Global Status Report on Alcohol*.

[2] Carlini E. L. A., Galduróz J. C. F., Noto A. R. (2007). *II Home Survey on the Use of Psychotropic Drugs in Brazil: Study Involving the 108 Major Cities of Brazil in 2005*.

[3] Carlini E. L. A., Noto A. R., Sanchez Z. V. D. M., Carlini C. M. A., Locatelli D. P. (2010). *VI National Survey on Consumption of Psychotropic Drugs Among Students of State-Owned and Private Schools in 27 Brazilian Capitals*.

[4] Lachenmeier D. W., Lima M. C. P., Nóbrega I. C. C. (2010). Cancer risk assessment of ethyl carbamate in alcoholic beverages from Brazil with special consideration to the spirits cachaca and tiquira. *BMC Cancer*.

[5] CNM—National Confederation of Cities (2012). *Deaths Caused by Use of Psychotropic Drug in Brazil*.

[6] Zacaroni L. M., Cardoso M. D. G., Saczk A. A. (2011). Analysis of organic contaminants and copper in cachaça. *Quimica Nova*.

[7] Alcarde A. R., Monteiro B. M. D. S., Belluco A. E. D. S. (2012). Chemical composition of sugar cane spirits fermented by different *Saccharomyces cerevisiae* yeast strains. *Quimica Nova*.

[8] Serafim F. A. T., da Silva A. A., Galinaro C. A., Franco D. W. (2012). Chemical profile comparison of sugarcane spirits from the same wine distilled in alembics and columns. *Quimica Nova*.

[9] Zapata J., Mateo-Vivaracho L., Cacho J., Ferreira V. (2010). Comparison of extraction techniques and mass spectrometric ionization modes in the analysis of wine volatile carbonyls. *Analytica Chimica Acta*.

[10] Baffa Junior J. C., Mendona R. C. S., Pereira J. M. D. A. T. K., Marques Pereira J. A., Soares N. D. F. F. (2011). Ethyl-carbamate determination by gas chromatography-mass spectrometry at different stages of production of a traditional Brazilian spirit. *Food Chemistry*.

[11] Riachi L. G., Santos A., Moreira R. F. A., de Maria C. A. B. (2014). A review of ethyl carbamate and polycyclic aromatic hydrocarbon contamination risk in cachaça and other Brazilian sugarcane spirits. *Food Chemistry*.

[12] Penteado J. C. P., Masini J. C. (2009). Heterogeneity of secondary alcohols in brazilian sugar cane spirits from diverse origins and processes of manufacture. *Quimica Nova*.

[13] Lachenmeier D. W., Sarsh B., Rehm J. (2009). The composition of alcohol products from markets in Lithuania and Hungary, and potential health consequences: a pilot study. *Alcohol and Alcoholism*.

[14] Lachenmeier D. W., Ganss S., Rychlak B. (2009). Association between quality of cheap and unrecorded alcohol products and public health consequences in Poland. *Alcoholism: Clinical and Experimental Research*.

[15] Plutowska B., Wardencki W. (2008). Determination of volatile fatty acid ethyl esters in raw spirits using solid phase microextraction and gas chromatography. *Analytica Chimica Acta*.

[16] de Souza P. P., Cardeal Z. D. L., Augusti R., Morrison P., Marriott P. J. (2009). Determination of volatile compounds in Brazilian distilled cachaça by using comprehensive two-dimensional gas chromatography and effects of production pathways. *Journal of Chromatography A*.

[17] Cortés S., Rodríguez R., Salgado J. M., Domínguez J. M. (2011). Comparative study between Italian and Spanish grape marc spirits in terms of major volatile compounds. *Food Control*.

[18] http://www.agenciaminas.mg.gov.br/.

[19] FGV—Getúlio Vargas Foundation (2008). *Estimation of Informality of Alcoholic Beverages in Brazil*.

[20] PNUD. United Nations Programme for Developing Countries Ranking do HDI of cities of Brazil. http://www.pnud.org.br/atlas.

[21] http://www.ibge.gov.br.

[22] Nappo S. A., Galduróz J. C. F. Psychotropic drug-related deaths in São Paulo City, Brazil.

[23] Lachenmeier D. W. (2007). Rapid quality control of spirit drinks and beer using multivariate data analysis of Fourier transform infrared spectra. *Food Chemistry*.

[24] Lachenmeier D. W., Rehm J., Gmel G. (2007). Surrogate alcohol: what do we know and where do we go?. *Alcoholism: Clinical and Experimental Research*.

[25] Lachenmeier D. W., Leitz J., Schoeberl K., Kuballa T., Straub I., Rehm J. (2011). Quality of illegally and informally produced alcohol in Europe: results from AMPHORA project. *Adicciones*.

[26] Lachenmeier D. W., Schoeberl K., Kanteres F., Kuballa T., Sohnius E.-M., Rehm J. (2011). Is contaminated unrecorded alcohol a health problem in the European Union? A review of existing and methodological outline for future studies. *Addiction*.

[27] Lachenmeier D. W., Haupt S., Schulz K. (2008). Defining maximum levels of higher alcohols in alcoholic beverages and surrogate alcohol products. *Regulatory Toxicology and Pharmacology*.

[28] Gallignani M., Ayala C., Brunetto M. D. R., Burguera J. L., Burguera M. (2005). A simple strategy for determining ethanol in all types of alcoholic beverages based on its on-line liquid-liquid extraction with chloroform, using a flow injection system and Fourier transform infrared spectrometric detection in the mid-IR. *Talanta*.

[29] Nóbrega I. C. C., Pereira J. A. P., Paiva J. E., Lachenmeier D. W. (2011). Ethyl carbamate in cachaa (Brazilian sugarcane spirit): extended survey confirms simple mitigation approaches in pot still distillation. *Food Chemistry*.

[30] Nóbrega I. C. C., Pereira J. A. P., Paiva J. E., Lachenmeier D. W. (2009). Ethyl carbamate in pot still cachaças (Brazilian sugar cane spirits): influence of distillation and storage conditions. *Food Chemistry*.

[31] Capobiango M., Oliveira E. S., Cardeal Z. L. (2013). Evaluation of methods used for the analysis of volatile organic compounds of sugarcane (*Cachaça*) and fruit spirits. *Food Analytical Methods*.

[32] Teixidó E., Moyano E., Santos F. J., Galceran M. T. (2008). Liquid chromatography multi-stage mass spectrometry for the analysis of 5-hydroxymethylfurfural in foods. *Journal of Chromatography A*.

[33] Jackson P., Fisher K. J., Attalla M. I. (2011). Tandem mass spectrometry measurement of the collision products of carbamate anions derived from CO_2_ capture sorbents: paving the way for accurate quantitation. *Journal of the American Society for Mass Spectrometry*.

[34] Rehm J., Mathers C., Popova S., Thavorncharoensap M., Teerawattananon Y., Patra J. (2009). Global burden of disease and injury and economic cost attributable to alcohol use and alcohol-use disorders. *The Lancet*.

[35] Rehm J., Kanteres F., Lachenmeier D. W. (2010). Unrecorded consumption, quality of alcohol and health consequences. *Drug and Alcohol Review*.

[36] Nagato L. A. F., Duran M. C., Caruso M. S. F., Barsotti R. C. F., Badolato E. S. G. (2001). Monitory of legality of alcoholic beverages samples analyzed in Adolfo Lutz Institute in São Paulo. *Ciência e Tecnologia de Alimentos*.

[37] Nuzhnyi V., Haworth A., Simpson R. (2004). Chemical composition, toxic, and organoleptic properties of noncommercial alcohol samples. *Moonshine Markets. Issues in Unrecorded Alcohol Beverage Production and Consumption*.

[38] Moreira R. F. A., Netto C. C., De Maria C. A. B. (2012). The volatile fraction of sugar cane spirits produced in Brazil. *Quimica Nova*.

[39] Caruso M. S. F., Nagato L. A. F., Alaburda J. (2010). Benzopyrene, ethyl carbamate and methanol in cachaça. *Quimica Nova*.

[40] Szücs S., Sárváry A., McKee M., Ádány R. (2005). Could the high level of cirrhosis in central and eastern Europe be due partly to the quality of alcohol consumed? An exploratory investigation. *Addiction*.

[41] Jiao Z., Dong Y., Chen Q. (2014). Ethyl carbamate in fermented beverages: presence, analytical chemistry, formation mechanism, and mitigation proposals. *Comprehensive Reviews in Food Science and Food Safety*.

[42] Aresta M., Boscolo M., Franco D. W. (2001). Copper(II) catalysis in cyanide conversion into ethyl carbamate in spirits and relevant reactions. *Journal of Agricultural and Food Chemistry*.

[43] Borges G. B. V., Gomes F. D. C. O., Badotti F., Silva A. L. D., Machado A. M. D. R. (2014). Selected *Saccharomyces cerevisiae* yeast strains and accurate separation of distillate fractions reduce the ethyl carbamate levels in alembic cachaças. *Food Control*.

[44] Mattová J., Poučková P., Kučka J. (2014). Chelating polymeric beads as potential therapeutics for Wilson’s disease. *European Journal of Pharmaceutical Sciences*.

[45] Capuano E., Fogliano V. (2011). Acrylamide and 5-hydroxymethylfurfural (HMF): a review on metabolism, toxicity, occurrence in food and mitigation strategies. *LWT—Food Science and Technology*.

